# Triglyceride-Glucose Index Linked to Hospital Mortality in Critically Ill Stroke: An Observational Multicentre Study on eICU Database

**DOI:** 10.3389/fmed.2020.591036

**Published:** 2020-10-28

**Authors:** Bingjun Zhang, Lingling Liu, Hengfang Ruan, Qiang Zhu, Dafan Yu, Yu Yang, Xuejiao Men, Zhengqi Lu

**Affiliations:** ^1^Department of Neurology, The Third Affiliated Hospital of Sun Yat-sen University, Guangzhou, China; ^2^Department of Hematology, The Third Affiliated Hospital of Sun Yat-sen University, Guangzhou, China

**Keywords:** triglyceride-glucose index, insulin resistance, stroke, mortality, ICU

## Abstract

**Objective:** The triglyceride-glucose (TyG) index is a reliable surrogate of insulin resistance and a marker for ischemic stroke (IS) incident. Whether the TyG index predicts stroke outcome remains uncertain. This study investigated the prognostic value of the TyG index in critically ill stroke patients.

**Methods:** This was a retrospective observational study that included stroke patients, and all data were extracted from the eICU Collaborative Research Database. The TyG index was calculated as the ln [fasting glucose level (mg/dL) × triglyceride level (mg/dL)/2]. Outcomes included the hospital and intensive care unit (ICU) death. Multivariate logistic regression was used to determine independent risk factors. The smoothing curves and forest plots were illustrated.

**Results:** A total of 4,570 eligible subjects were enrolled. The mean level of TyG index was 9.1 ± 0.7. The hospital and ICU mortality rate were 10.3 and 5.0%, respectively. TyG index as a continuous variable was associated hospital mortality in univariate analysis (OR 1.723, 95% CI 1.524–1.948, *P* < 0.001), adjusted model 1 (OR 1.861, 95% CI 1637–2.116, *P* < 0.001), and adjusted model 2 (OR 2.543, 95% CI 1.588–4.073, *P* < 0.001). TyG was also associated ICU mortality in univariate analysis (OR 2.146, 95% CI 1.826–2.523, *P* < 0.001), adjusted model 1 (OR 2.183, 95% CI 1.847–2.580, *P* < 0.001), and adjusted model 2 (OR 2.672, 95% CI 1.376–5.188, *P* < 0.001). The smoothing curves observed a continuous linear association after adjusting all covariates both in hospital and ICU mortality. Subgroup analysis demonstrated TyG index was associated with increased risk of hospital and ICU death in critically ill IS (*P* < 0.05), but not in hemorrhage stroke (*P* > 0.05).

**Conclusion:** The TyG index is a potential predictor for hospital and ICU mortality in critically ill stroke patients, especially in IS patients.

## Introduction

Stroke is a leading cause of mortality and disability worldwide. According to global burden of disease (GBD) 2016 stroke surveys, it was estimated that there were 5.5 million deaths due to stroke, and the global lifetime risk of stroke from the age of 25 years onward was ~25% (1, 2). The economic costs of treatment and post-stroke care are substantial, while the global burden of stroke has been increasing. Furthermore, an increasing number of stroke patients, who have higher hospital mortality, are being admitted to an intensive care unit (ICU) for management of severe neurological impairment and post-stroke complications (3, 4). Therefore, it is still important to identify the controllable risk factors targeted by preventive strategies and health-care management that lead to a larger decrease in stroke mortality.

The triglyceride-glucose (TyG) index combines both levels of fasting plasma glucose and triglyceride, and it has been reported to be significantly correlated with insulin resistance (IR) and to be a reliable surrogate marker of IR (5). Previous studies have indicated that the TyG index is associated with cardiovascular disease morbidity and mortality in the general population and different types of patient cohorts (6–9). Lately, the TyG index has been proposed as a direct marker for the risk of incident ischemic stroke (IS) in general population (10). To date, no relevant study has focused on the impact of the TyG index on mortality in patients with critically ill stroke including IS and hemorrhage stroke (HS). Therefore, this study aimed to specifically investigate the association between TyG index and mortality of stroke in a large observational multicentre study on eICU database.

## Materials and Methods

### Data Source

Data were collected from the eICU Collaborative Research Database v2.0. The database was a multi-center ICU database for over 200,000 admissions in 2014 and 2015 at 208 United States hospitals (11). The eICU database included hourly physiological readings from bedside monitors, records of demographic characteristics, severity of illness measures, diagnoses, treatment, and other clinical data collected during routine medical care. The use of this database has been approved by the institutional review boards of Massachusetts Institute of Technology (Cambridge, MA, USA). One author (Bingjun Zhang) obtained the access and was responsible for the data extraction (certification number: 36492956).

### Participants

Patients with a primary diagnosis of brain stroke, recorded on patient dataset, were potentially eligible. The stroke patients can be grouped into 4 categories: IS group, HS group, IS with HS group, and unknown/others group. HS group included subarachnoid hemorrhage and intracerebral hemorrhage patients.

### Demographic and Laboratory Variables

The following data were extracted from the eICU database: gender, age, ethnicity, admission height, admission weight, stroke types, plasma glucose values, blood lipids values, acute physiology, and chronic health evaluation (APACHE) IV score, the status of discharge hospital or ICU, in-hospital length of stay (LOS), ICU LOS, comorbidities, mechanical ventilation. The APACHE IV system is a tool used to risk-adjust ICU patients for ICU performance benchmarking (12), and it provides estimates of the probability that a patient dies given data from the first 24 h after the ICU admission (11).

If glucose, lipids, and some variables were measured for several times after the ICU entry, data of the first time were used. The TyG index was calculated as the ln [fasting glucose level (mg/dL) × triglyceride level (mg/dL)/2].

The primary outcome was hospital mortality, with ICU mortality as a secondary outcome.

Variables with more than 10% missing values were excluded from the analysis. Single imputation of EM algorithm was performed for variables with missing values of <10%.

### Statistical Analyses

Categorical variables were expressed in absolute number with percentage and analyzed by chi-square or Fisher exact test. Continuous variables were first assessed for normality. Normal data were expressed in mean with standard deviation (SD) and compared using Student *t*-test or one-way ANOVA. Non-normal data were expressed in median with interquartile range (IQR) and were compared using Wilcoxon rank-sum test. Variables with two-tailed *p* value < 0.05 were considered to be statistically significant and were included in the regression model. Multivariate-adjusted odds ratios (OR) and 95% confidence intervals (CI) for the study outcomes and TyG index (1 unit and quartile) were calculated by logistic regression analysis. The multivariate model 1 included gender, age, ethnicity, while model 2 included model 1 plus stroke types, coronary heart disease, diabetes mellitus, heart failure, plasma glucose, total cholesterol (TC), low-density lipoprotein-cholesterol (LDL-C), triglyceride, mechanical ventilation, APACHE IV score, LOS. All of the analyses were performed using the SPSS version 26 (Chicago, Illinois). All *p* values were two-tailed and a *p* value < 0.05 was considered statistically significant. The smoothing curves and forest plots were illustrated by EmpowerStats (X&Y Solutions, Inc., Boston, MA).

## Results

The initial search identified 200,859 ICU admissions from the eICU database. A total of 6,849 subjects with a primary diagnosis of brain stroke were identified. A total of 2,279 subjects were excluded because they did not have mortality values. The final cohort included 4,570 patients, including 4,101 (89.7%) survivors before hospital discharge and 4,341 (95.0%) survivors before ICU discharge. The study patients had an average age of 66.3 ± 14.2 years and 2,358 (51.6%) patients were male. The mean level of TyG index was 9.1 ± 0.7. The hospital and ICU mortality rate were 10.3% and 5.0%, respectively.

### Baseline Characteristics

The baseline characteristics between survivors and non-survivors groups are described in Table 1. Hospital non-survivor group had higher age, plasma glucose, triglyceride, ICU LOS than hospital survivor group (*P* < 0.05), while lower TC and LDL-C (*P* < 0.05). There was no significant difference on sex, ethnicity, height, weight, HDL-C, hypertension, atrial fibrillation, hospital LOS (*P* > 0.05). Furthermore, hospital non-survivor group had more frequent diabetes mellitus, coronary heart disease, heart failure, mechanical ventilation than hospital survivor group (*P* < 0.05). Stroke type was significantly different between the two groups (*P* < 0.05). Patients in the hospital non-survivor group had higher APACHE IV score (70.8 ± 25.0 vs. 47.7 ± 19.0, *P* < 0.001) and TyG index (9.3 ± 0.7 vs. 9.0 ± 0.7, *P* < 0.001) than those survivor group.

**Table 1:** Baseline clinical and laboratory characteristics of the study patients.

**Variables**		**Hospital mortality**			**ICU mortality**	
	**Non-survivors *n* = 469**	**Survivors *n* = 4,101**	***P***	**Non-survivors *n* = 229**	**Survivors *n* = 4,341**	***P***
Age, years	69.2 ± 13.3	66.3 ± 14.0	<0.001	65.8 ± 14.1	66.7 ± 14.0	0.336
Male, n (%)	237 (50.5)	2121 (51.7)	0.837	116 (50.7)	2242 (51.6)	0.932
Ethnicity, n (%)			0.172			0.868
Caucasian	341 (72.7)	3059 (74.6)		170 (74.2)	3230 (74.4)	
African American	49 (10.4)	452 (11.0)		25 (10.9)	476 (11.0)	
Native American	1 (0.2)	24 (0.6)		0	25 (0.6)	
Hispanic	40 (8.5)	229 (5.6)		14 (6.1)	255 (5.9)	
Asian	9 (1.9)	89 (2.2)		4 (1.7)	94 (2.2)	
Other/unknown	29 (6.2)	248 (6.0)		16 (7.0)	261 (6.0)	
Admission height (cm)	168.4 ± 13.3	168.9 ± 15.6	0.514	168.8 ± 11.2	168.8 ± 15.6	0.966
Admission weight (kg)	81.0 ± 21.9	82.7 ± 22.4	0.108	82.4 ± 24.1	82.6 ± 22.3	0.9
Stroke types, n (%)			<0.001			<0.001
IS	201 (42.9)	1,184 (45.9)		92 (40.2)	1,993 (45.9)	
HS	174 (37.1)	907 (22.1)		89 (38.9)	992 (22.9)	
IS with HS	27 (5.8)	109 (2.7)		17 (7.4)	119 (2.7)	
Unknown/others	67 (14.3)	1201 (29.3)		31 (13.5)	1237 (28.5)	
Hypertension, n (%)	169 (36.0)	1,377 (33.6)	0.287	75 (32.8)	1,471 (33.9)	0.723
Diabetes mellitus, n (%)	75 (16.0)	480 (11.7)	0.007	37 (16.2)	518 (11.9)	0.056
Coronary heart disease, n (%)	42 (9.0)	214 (5.2)	0.001	23 (10.0)	233 (5.4)	0.003
Atrial fibrillation, n (%)	62 (13.2)	463 (11.3)	0.214	31 (13.5)	494 (11.4)	0.318
Heart failure, n (%)	24 (5.1)	120 (2.9)	0.01	15 (6.6)	129 (3.0)	0.003
Plasma glucose (mg/dl)	211 ± 95.8	164.6 ± 80.7	<0.001	230.7 ± 112	166.2 ± 80.5	<0.001
TC (mg/dL)	156.2 ± 47.1	163.2 ± 45.7	0.002	159.4 ± 48.1	162.7 ± 45.8	0.291
LDL-C (mg/dL)	84.3 ± 37.9	92.4 ± 38.3	<0.001	86.4 ± 38.6	91.8 ± 38.3	0.037
HDL-C (mg/dL)	45.2 ± 17.9	45.5 ± 15.7	0.775	44.2 ± 19.3	45.5 ± 15.8	0.216
Triglyceride (mg/dL)	138.5 ± 95.5	129.9 ± 94.7	0.064	151.9 ± 102.1	129.7 ± 94.3	0.001
Mechanical ventilation, n (%)	387 (82.5)	2,217 (54.1)	<0.001	202 (88.2)	2,402 (55.3)	<0.001
APACHE IV score	70.8 ± 25.0	47.7 ± 19.0	<0.001	70.8 ± 25.0	47.7 ± 19.0	<0.001
Hospital LOS, days	5.8 (3.1, 10.2)	5.8 (3.3, 9.0)	0.766	4.6 (2.6, 8.7)	5.8 (3.4, 9.1)	<0.001
ICU LOS, days	3.7 (2.0, 6.8)	2.0 (1.2, 3.7)	<0.001	3.9 (2.3, 7.1)	2.0 (1.2, 3.8)	<0.001
TyG index	9.3 ± 0.7	9.0 ± 0.7	<0.001	9.3 ± 0.7	9.0 ± 0.7	<0.001

*ICU, intensive care unit; IS, ischemic stroke; HS, hemorrhage stroke; TC, total cholesterol; LDL-C, low-density lipoprotein-cholesterol; HDL-C, high-density lipoprotein-cholesterol; APACHE, acute physiology and chronic health evaluation; LOS, length of stay, TyG triglyceride-glucose*.

In addition, there was no significant difference on age and TC between ICU survivors and ICU non-survivors groups (*P* > 0.05). ICU non-survivor group had lower hospital LOS than ICU survivor group (*P* < 0.001). Patients in the ICU non-survivor group had higher APACHE IV score (70.8 ± 25.0 vs. 47.7 ± 19.0, *P* < 0.001) and TyG index (9.3 ± 0.7 vs. 9.0 ± 0.7, *P* < 0.001) than those survivor group. Other results in ICU groups were consistent with those in-hospital groups.

### TyG Index and Mortality of Critically Ill Stroke

Univariate and multivariate logistic regression revealed the association between TyG index and critically ill stroke mortality (Table 2). In univariate analysis, TyG index as a continuous variable was associated hospital mortality (OR 1.723, 95% CI 1.524–1.948, *P* < 0.001). Several risk factors including important clinical and significant variables in the univariate model were included in the multivariate model for adjustment, and the TyG index also remained to be an independent predictor of hospital mortality in adjusted model 1 (OR 1.861, 95% CI 1637–2.116, *P* < 0.001) and model 2 (OR 2.543, 95% CI 1.588–4.073, *P* < 0.001). Furthermore, TyG index was also associated ICU mortality in univariate analysis (OR 2.146, 95% CI 1.826–2.523, *P* < 0.001), adjusted model 1 (OR 2.183, 95% CI 1.847–2.580, *P* < 0.001), and adjusted model 2 (OR 2.672, 95% CI 1.376–5.188, *P* < 0.001).

**Table 2:** Logistic regression of TyG index for mortality in all study groups.

		**Univariate**		**Model 1**		**Model 2**	
	**TyG index**	**OR (95% CI)**	***p***	**OR (95% CI)**	***p***	**OR (95% CI)**	***p***
**Hospital mortality**	Per 1-unit increase	1.723 (1.524–1.948)	<0.001	1.861 (1.637–2.116)	<0.001	2.543 (1.588–4.073)	<0.001
	Quartile 1	Reference		Reference		Reference	
	Quartile 2	1.635 (1.170–2.283)	0.004	1.664 (1.190–2.326)	0.003	1.483 (1.030–2137)	0.034
	Quartile 3	2.294 (1.669–3.153)	<0.001	2.373 (1.723–3.268)	<0.001	2.018 (1.383–2.946)	<0.001
	Quartile 4	3.486 (2.572–4.724)	<0.001	3.838 (2.819–5.224)	<0.001	2.546 (1.547–4.191)	<0.001
	P for trend		<0.001		<0.001		<0.001
**ICU mortality**	Per 1-unit increase	2.146 (1.826–2.523)	<0.001	2.183 (1.847–2.580)	<0.001	2.672 (1.376-5.188)	<0.001
	Quartile 1	Reference		Reference		Reference	
	Quartile 2	1.977 (1.130–3.459)	0.017	1.973 (1.127–3.452)	0.017	1.521 (0.827-2.797)	0.177
	Quartile 3	3.564 (2.123–5.982)	<0.001	3.573 (2.127–6.002)	<0.001	2.513 (1.377-4.586)	0.003
	Quartile 4	6.173 (3.764–10.126)	<0.001	6.193 (3.767–10.180)	<0.001	2.826 (1.341-5.956)	0.006
	P for trend		<0.001		<0.001		<0.001

Model 1 adjusted for gender, age, ethnicity.

Model 2 adjusted for model 1 plus stroke types, coronary heart disease, diabetes mellitus, heart failure, plasma glucose, TC, LDL-C, triglyceride, mechanical ventilation, APACHE IV score, LOS.

*ICU, intensive care unit; TyG, triglyceride-glucose; OR, odds ratios; CI, confidence intervals; TC, total cholesterol; LDL-C, low-density lipoprotein-cholesterol; APACHE, acute physiology and chronic health evaluation; LOS, length of stay*.

**Figure 1:**
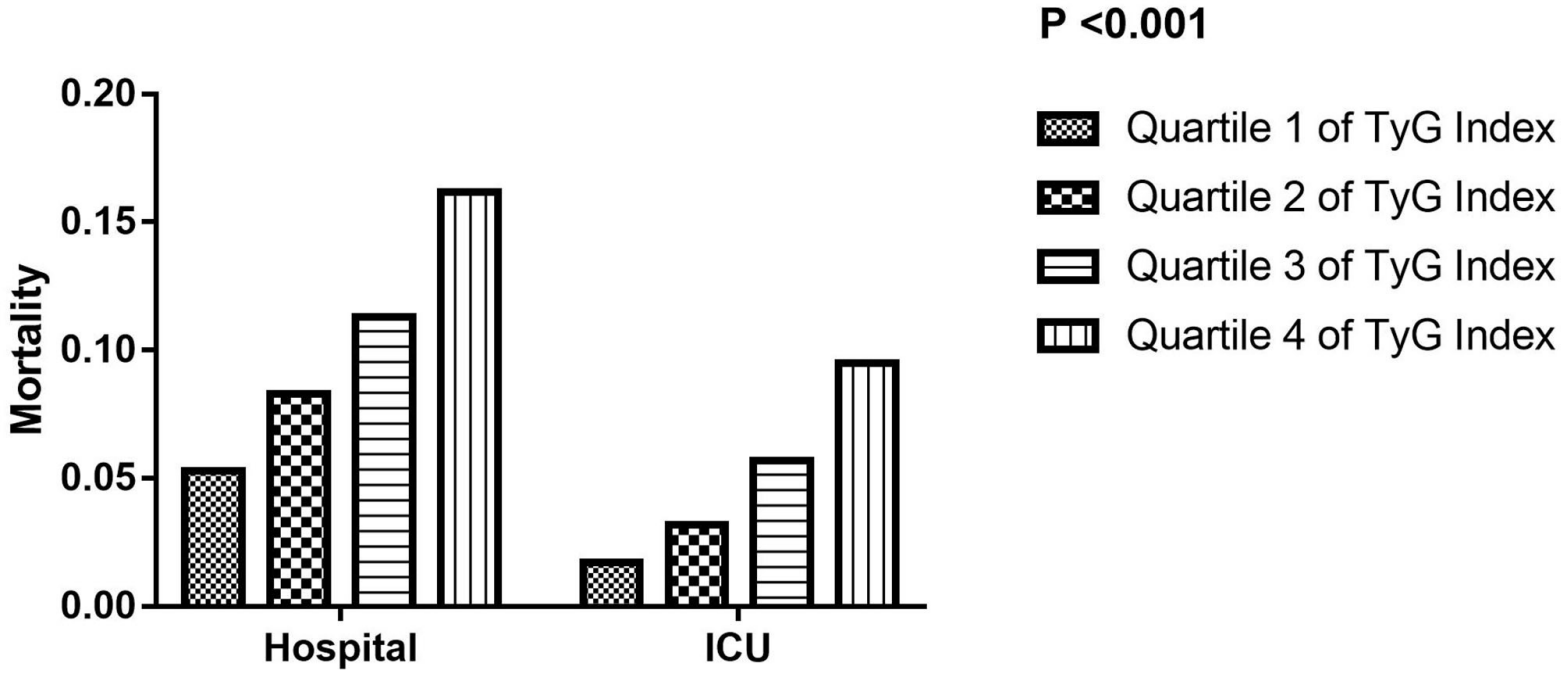
The hospital and ICU mortality according to TyG index quartiles. ICU, intensive care unit; TyG, triglyceride-glucose.

In addition, all participants were stratified into four groups based on the quartile of TyG index. The mean levels of TyG index were 8.3 ± 0.3, 8.8 ± 0.1, 9.2 ± 0.1, and 10.0 ± 0.1, respectively. The hospital and ICU mortality significantly increased with increasing quartiles of the TyG index (*P* < 0.001) (Figure 1). When dividing TyG index into quartiles, we observed a quartile increment in TyG index was associated with the increased hospital and ICU mortality of critically ill stroke in univariate, adjusted model 1 and adjusted model 2 logistic regression analysis, with a significant trend across the quartiles (*P* for trend < 0.001, Table 2).

**Figure 2:**
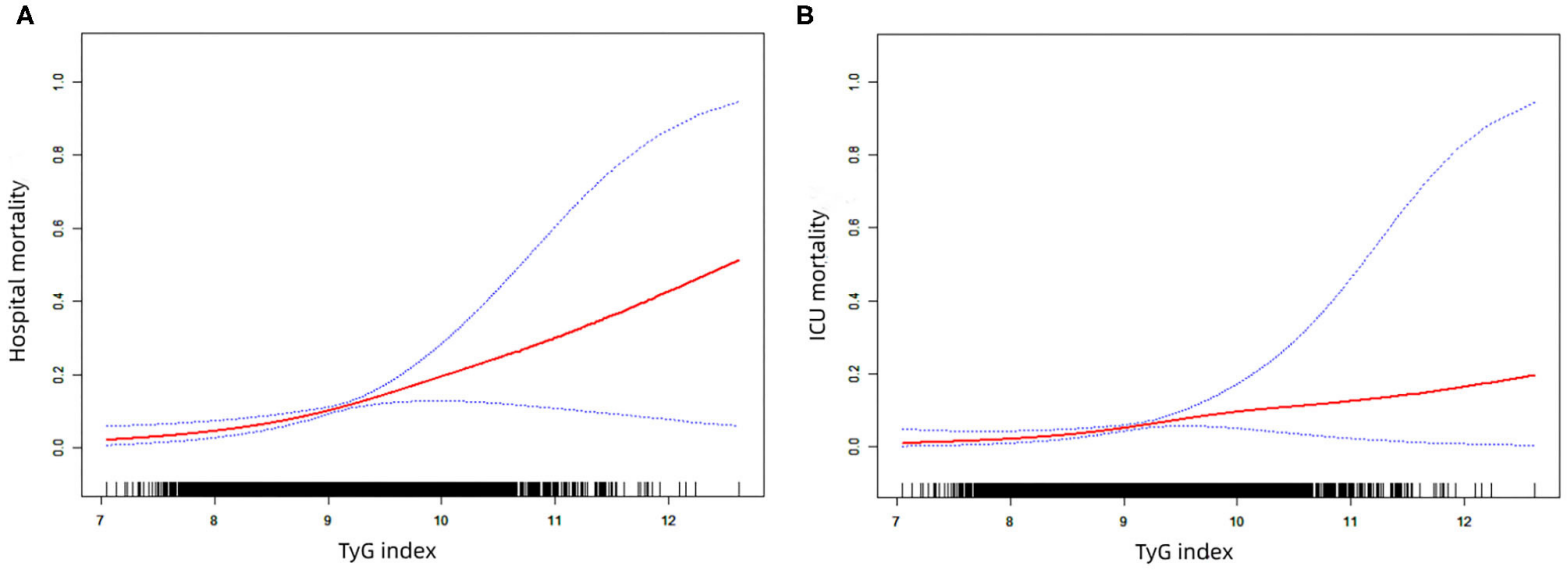
The smoothing curves of the hospital **(A)** and ICU **(B)** mortality of critically ill stroke against TyG index. ICU, intensive care unit; TyG triglyceride-glucose.

To further explore the relationship between TyG index and the mortality of critically ill stroke, we plotted the smoothing curves of TyG index against the hospital and ICU mortality of critically ill stroke (Figure 2). In this plot, we observed a continuous linear association after adjusting all covariates both in hospital (Figure 2A) and ICU (Figure 2B) mortality. This finding was consistent with the stepwise increased OR in the analysis of multivariate logistic regression.

### Relationship of TyG Index to Mortality According to Stroke Types

**Figure 3:**
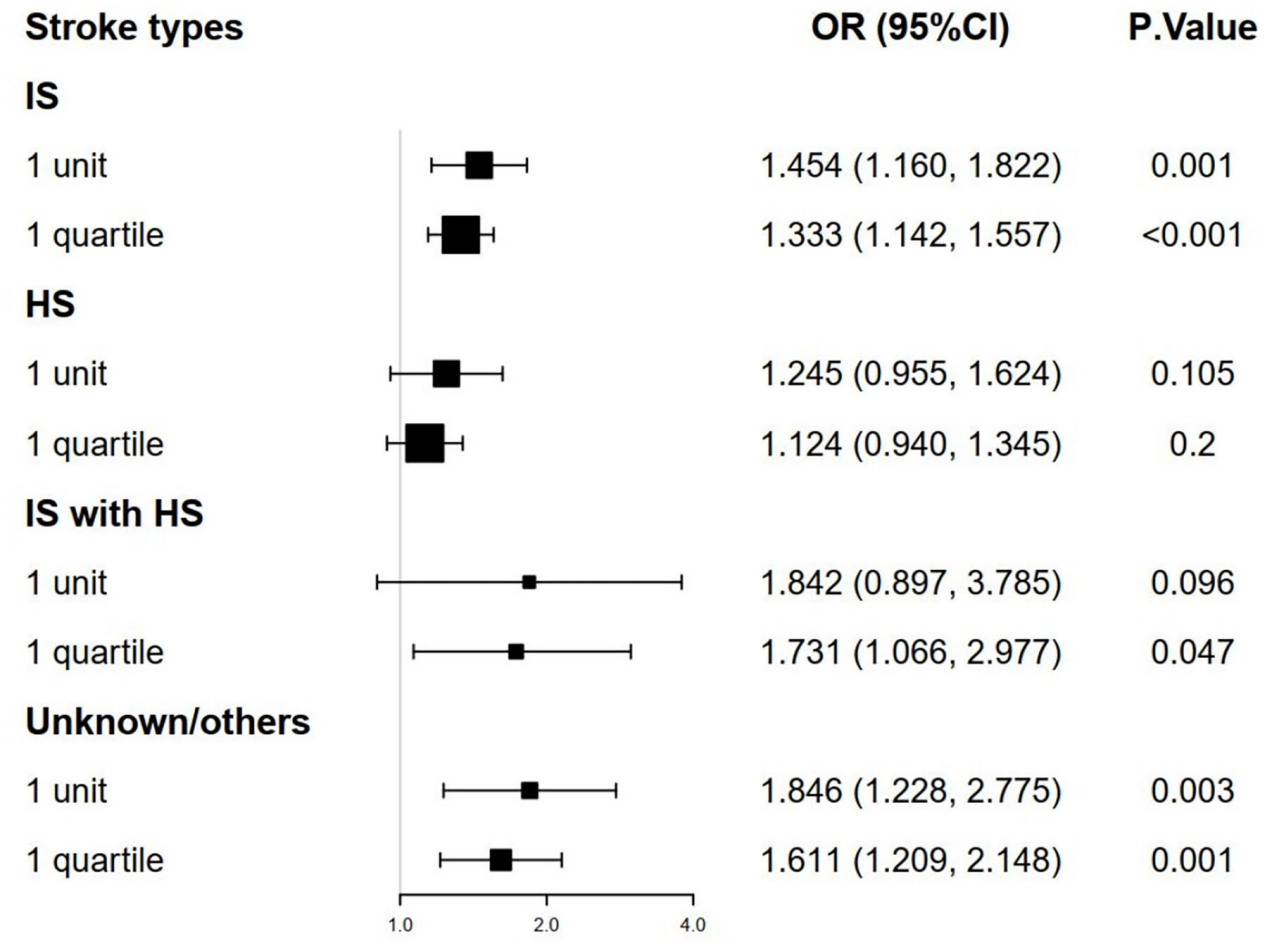
The forest plot of the hospital mortality of critically ill stroke according to stroke types and TyG index. TyG, triglyceride-glucose.

**Figure 4:**
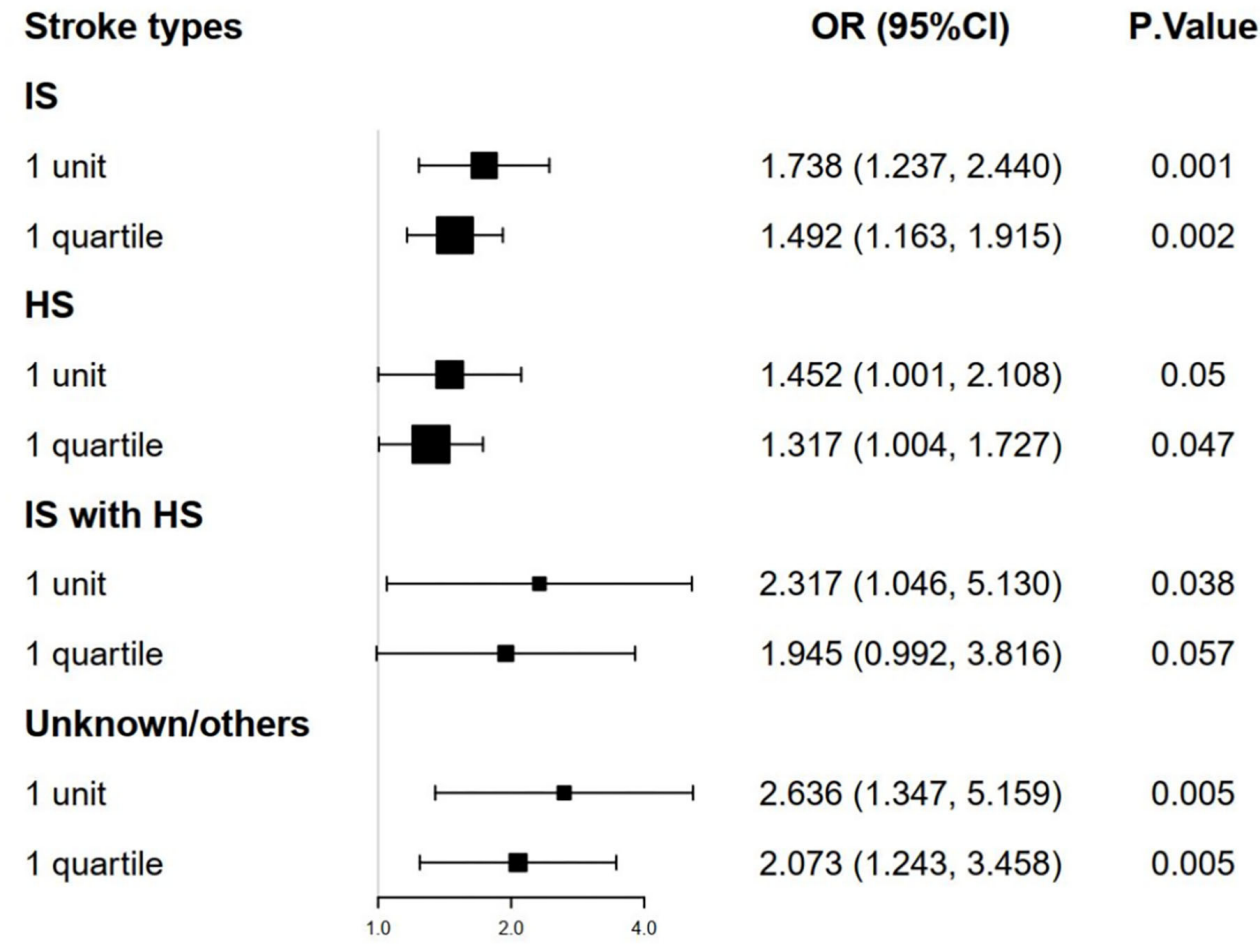
The forest plot of the ICU mortality of critically ill stroke according to stroke types and TyG index. ICU, intensive care unit; TyG triglyceride-glucose.

The stroke patients were grouped into 4 types: IS group, HS group, IS with HS group, and unknown/others group. Multivariate logistic regression models were analyzed to identify the association between TyG index and mortality according to the stroke type and TyG index. We found that a higher TyG index was significantly associated with the increased hospital and ICU mortality in IS group and unknown/others group (Figures 3, 4, adjusted *P* < 0.05). However, the similar result did not occur in HS group and IS with HS group (Figures 3, 4, adjusted *P* > 0.05).

## Discussion

To the best of our knowledge, this is the first study to evaluate the association of the TyG index with hospital and ICU mortality in critically ill stroke patients. The main findings are as follows: (1) the TyG index is an independent predictor for hospital and ICU mortality in patients with critically ill stroke; (2) the hospital and ICU mortality correlated proportionally with the increment of TyG index, implicating the linearity of TyG index as an indicator of critically ill stroke; (3) subgroup analysis demonstrated TyG index was associated with increased risk of hospital and ICU death in critically ill IS, but not in HS.

The TyG index, as the product of fasting plasma glucose and triglyceride, is a novel index that has been well-recognized as a simple and reliable surrogate of IR (13). The homeostatic model assessment of IR (HOMA-IR) has been traditionally used to estimate IR (14). However, insulin levels must be required to calculate the HOMA-IR index. Compared with the inconvenient HOMA-IR, the TyG index does not require levels of insulin and may apply to all of the patients and healthy population. Recent studies indicated that the TyG index has been shown to be superior to HOMA-IR in predicting IR (15). Furthermore, several studies conducted in Asia and Europe validated the strong association between TyG and incidence of diabetes mellitus (13, 16). Won et al. reported the TyG index was independently associated with arterial stiffness in a relatively healthy Korean population (17). Other studies demonstrated the TyG index was an independent predictor of coronary artery calcification progression and risk of cardiovascular diseases (8, 18). In addition, previous studies suggested the TyG index predicted severity and outcomes in patients with acute coronary syndrome (6, 19, 20). Wang et al. reported the TyG index predicted future cardiovascular events in patients with diabetes and acute coronary syndrome independently of known cardiovascular risk factors (7). However, in a previous study based on a Caucasian population, the TyG index displayed an insignificant association with stroke (21). Later, another study based on the same Caucasian cohort, identified metabolic health and obesity states groups based on TyG index was significantly associated with the risk of IS (22). Additionally, a recently published epidemiological investigation expanded the use of TyG index as a direct marker for the risk of IS (10). Zhou et al. demonstrated that the baseline TyG index was significantly associated with an increased risk of 12-month all cause mortality without accelerating poor functional outcome in IS patients (23). However, no relevant study focus on the association between TyG index and outcome in patients with critically ill stroke. The present study investigated the relationship between the TyG index and the hospital mortality in critically ill stroke on eICU database. We found that the TyG index was significantly associated with mortality in ICU stroke after adjusting for confounding factors. Furthermore, the TyG index had a continuous linear correlation with the mortality of ICU stroke.

The mechanism underlying the relationship between the TyG index and stroke is not fully elucidated. The formula of TyG index is composed of glucose and triglyceride, while recent evidence has proved that glucose and triglyceride disorders are the risk factors of stroke (24, 25). Hyperglycemia in stroke patients is associated with increased infarct volume, higher risk of secondary hemorrhagic transformation, and reduced recanalization rates after intravenous thrombolysis and endovascular thrombectomy (26, 27). Hyperglycemia on admission is strongly predictive of unfavorable outcome and high mortality (28). Under experimental stroke conditions, hyperglycemia promotes tissue acidosis, the production of reactive oxygen and nitrogen species, inflammatory cell infiltration, and the imbalance of matrix metalloprotease-9 that increase infarct size, brain swelling, hemorrhagic transformation, blood–brain barrier disruption and results in more severe neurological deficits (26, 29). A number of studies have indicated that hypertriglyceridemia is associated with early neurological deterioration and mortality in patients with stroke (30). Hypertriglyceridemia is related to the development of atherosclerosis and the loss of reserve capacity of cerebral vessels (31). Triglyceride, which comprises large amounts of arachidonic acid, can be involved in generating oxidative stress and thrombus (32). Hyperglycemia and dyslipidemia are two basic hallmarks of IR, while IR may play a vital role in the development of hyperglycemia and dyslipidemia (23). The TyG index has been deemed as a useful atherogenic indicator linked to IR and metabolic syndrome. IR may be the mechanism in increasing mortality in stroke. Firstly, IR may increase proinflammatory cytokines and enhance prothrombotic responses, thus exacerbating damage in the brain after stroke (33, 34). Secondly, IR may cause sympathetic activity and catabolism in muscles, thus enhancing muscle loss and leading to poor functional outcomes (35). Thirdly, IR may increase platelet adhesion, enhance atherosclerosis progression, facilitate plaque instability, and therefore contribute to severity of stroke, via promoting apoptosis of vascular smooth muscle cells macrophages, and endothelial cells (36, 37). Fourthly, IR may augment the role of modifiable risk factors of stroke, such as hypertension, atrial fibrillation (10). Although the present study had showed the association between TyG index and post-stroke outcomes, the underlying molecular mechanisms involved in this association should be further investigated in the future study.

Stroke accounts for almost 5% of all disability-adjusted life-years and 10% of all deaths worldwide (2). From different epidemiological surveys, stroke mortality in different periods varies from 5 to 72% (38, 39). Stroke mortality has been declining since the early twentieth century (40). The reasons for this are not completely understood, although the decline is welcome (40). However, due to the increasing complexity of stroke treatment and severe conditions, an increasing proportion of acute stroke patients are being admitted to an ICU (41, 42). A study including 4,958 consecutive stroke patients reported 347 (7.0%) patients required ICU admission at any time point during their index hospitalization (4). In-hospital mortality of ICU stroke reported in the literature varies widely, highly depending on the patient characteristics. A previous small study found the mortality was 38.7% (43/111) in stroke patients requiring ICU admission (41). An United States study including 448 ICU stroke patients provided hospital mortality was 30% (43). Recently, a prospective observation trial reported the mortality was 7.5% in critically ill stroke patients (44). These patients with high hospital mortality mainly associated with older age, poor neurological severity at admission, high APACHE score, impaired consciousness, intracranial hemorrhage, and need for mechanical ventilation (41, 42). Moreover, functional outcomes in survivors appear to be poor (45). The current study based on eICU database, indicated the hospital and ICU mortality were 10.3 and 5.0%, respectively. Furthermore, we found a new risk factor, the TyG index, for the mortality of critically ill stroke.

In subgroup analysis, we demonstrated TyG index was associated with increased risk of hospital and ICU death in critically ill IS, but not in HS. In agreement with previous GBD 2016 report, we did not estimate the mortality due to subarachnoid hemorrhage and intracerebral hemorrhage separately (1). Of the total number of prevalent strokes, over 80% were IS. However, the number of global deaths due to IS was slightly lower than the number due to HS deaths (1). In contrast, a previous neurology ICU study demonstrated HS was more frequent than IS (71.9 vs. 28.1%) (46). Several studies, including our present study, described TyG index was a useful marker in IS. However, to our knowledge, no relevant study has evaluated the relationship between the TyG index and HS. Although previous studies had focused on the relationship between IR and HS, the results had been discrepancy. The Rotterdam study and the Uppsala study had previously examined the association between IR and risk of HS, finding virtually no evidence of an association (47, 48). Later, a large United States stroke cohort showed IR may be a protective effect on HS (49). In the current study, we did not observe a significant association of the TyG index with all-cause death in critically ill HS in either unadjusted or adjusted analysis.

Although our study based on a large multicentre critical care database, it still has some limitations. First, this was a retrospective analysis derived from an observational study, which could not definitively establish causality. Second, the eICU v2.0 did not contain the data on head imaging, neurological severity scores, and follow-up outcomes after discharge. Residual confounding could exist. Third, the baseline levels of plasma glucose and triglyceride could be affected by the use of antidiabetic and lipid-lowering drugs before ICU admission. The TyG index might have changed during hospital; therefore, it is unknown whether the change in the TyG index could have predicted the mortality. Fourth, we did not measure HOMA-IR because the examination of insulin levels is not included in the eICU v2.0. Last, the data were from the United States, and thus the results may not apply fully to ICUs elsewhere with different practices or resources.

## Conclusions

In this multicentre critically ill stroke cohort, we observed the association of the TyG index with hospital and ICU mortality in stroke patients. For the first time, this study demonstrated that the TyG index is a potential predictor for hospital and ICU mortality in critically ill stroke patients, especially in IS patients. Furthermore, the TyG index has a linear correlation with the mortality of ICU stroke. Most importantly, these findings suggested that the TyG index may be a useful indicator for risk stratification and prognosis in patients with critically ill stroke. Further prospective studies are required to confirm our findings.

## Data Availability Statement

Data were fully available at https://eicu-crd.mit.edu/. Source code for data extraction can be found at https://github.com/tyg999/TyG-stroke.

## Ethics Statement

The establishment of this database was approved by the Massachusetts Institute of Technology (Cambridge, MA), and consent was obtained for the original data collection. Therefore, the ethical approval statement and the need for informed consent were waived for this manuscript.

## Author Contributions

BZ, LL, HR, and ZL designed research. QZ and DY performed experiments and analyzed data. BZ, YY, and XM wrote the main manuscript text and prepared figures. BZ and XM edited and revised manuscript. All authors reviewed and approved the manuscript.

## Conflict of Interest

The authors declare that the research was conducted in the absence of any commercial or financial relationships that could be construed as a potential conflict of interest.
